# Biomechanical and clinical evaluation of interlocking hip screw in Pauwels Ⅲ femoral neck fractures: A comparison with inverted triangle cannulated screws

**DOI:** 10.3389/fbioe.2022.1047902

**Published:** 2022-10-31

**Authors:** Jian Zhang, Haozheng Jiang, Wei Dai, Salad Abdirahman Hersi, John Chun tien chui wan Cheong, Zhenchen Chu, Zhiyuan Lou, Deqiang Zhang, Changjian Liu, Kang Tian, Xin Tang

**Affiliations:** ^1^ Department of Orthopedics, First Affiliated Hospital of Dalian Medical University, Dalian, China; ^2^ Graduate School of Dalian Medical University, Dalian Medical University, Dalian, China

**Keywords:** biomechanics, osteotomy, osteosynthesis, internal fixation, femoral neck fracture, cannulated screws

## Abstract

**Purpose:** To compare biomechanical and clinical properties of the novel internal fixation Interlocking Hip Screw (IHS) and conventional inverted triangle cannulated screws (ITCS) for treatment of Pauwels Ⅲ femoral neck fractures.

**Methods:** Twenty synthetic femurs were osteotomized to simulate 70° Pauwels Ⅲ femoral neck fractures and randomly divided into two groups: Group IHS and Group ITCS. Specimens were loaded in quasi-static ramped and cyclical compression testing in 25° adduction to analyze for axial stiffness, failure load, and interfragmentary displacement. 21 matched patients with Pauwels Ⅲ femoral neck fracture who received closed reduction and internal fixation from January 2020 to January 2021 in both Group IHS and Group ITCS. Demographic data, time to surgery, operating duration, intraoperative blood loss, number of fluoroscopies, length of hospital stay, fracture healing time, Harris Hip Score (HHS), the score of Visual Analogue Scale (VAS) and complications such as nonunion, avascular necrosis, and femoral neck shortening were compared.

**Results:** All specimens in the two groups survived in the axial and cyclical compression test. The axial stiffness was significantly higher for Group IHS (277.80 ± 26.58 N/mm) *versus* Group ITCS (205.33 ± 10.46 N/mm), *p* < 0.05. The maximum failure loading in Group IHS performed significantly higher than in Group ITCS (1,400.48 ± 71.60 N *versus* 996.76 ± 49.73 N, *p* < 0.05). The interfragmentary displacement of the cyclic loading test for Groups IHS and Group ITCS was 1.15 ± 0.11 mm and 1.89 ± 0.14 mm, respectively, *p* < 0.05. No significant difference was found in terms of demographic data, time to surgery, intraoperative blood loss, length of hospital stay and the occurrence of nonunion and avascular necrosis between groups. Shorter operating duration and fewer intraoperative fluoroscopic views were noticed using IHS compare to ITCS, *p* < 0.05. The HHS was 72.14 ± 5.76 and 86.62 ± 5.01 in Group IHS, and was 67.29 ± 5.27 and 81.76 ± 5.13 in Group ITCS at 3-month and 6-month follow-up, respectively, *p* < 0.05. The magnitude of femoral neck shortening was significantly lower in Group IHS compared to Group ITCS (4.80 ± 1.03 mm *versus* 5.56 ± 1.21 mm, *p* < 0.05).

**Conclusion:** Our study demonstrated that IHS provided better biomechanical and clinical performance due to its unique biological and biomechanical mechanisms, compared with ITCS. Thus, IHS is a feasible alternative to ITCS for the fixation of Pauwels Ⅲ femoral neck fractures.

## 1 Introduction

Hip fractures cripple 4.5 million individuals worldwide each year, of which femoral neck fractures account for about 53%. More than half of hip fractures will occur in Asia by 2040, accompanied by an enormous socioeconomic burden and medical challenge (Bhandari and Swiontkowski, 2017). Most femoral neck fractures in young adults are Pauwels Ⅲ femoral neck fractures caused by high energy trauma with a high risk of complications such as nonunion, avascular necrosis, and femoral neck shortening (Jiang et al., 2021). A series of implants have been proposed for superior prognosis of Pauwels Ⅲ femoral neck fractures (Stoffel et al., 2017; Chan, 2019; Duffin and Pilson, 2019), however, the optimal choice of internal fixation is still controversial and no consensus has been established (Florschutz et al., 2015). Inverted triangle cannulated screws (ITCS) were widely used for Pauwels Ⅲ femoral neck fractures in China (Slobogean et al., 2017). Nowadays, the novel minimally invasive implant Interlocking Hip Screw (IHS) consisted of a small side plate and the dynamic combined interlocking screws had made preliminary progress in the clinical application for the Pauwels Ⅲ femoral neck fractures. To the authors’ knowledge, limited biomechanical and clinical outcomes data exist on this new device. Therefore, the goal of our study is to compare both the biomechanical and clinical properties of IHS and ITCS in the fixation of Pauwels Ⅲ femoral neck fractures.

## 2 Materials and methods

### 2.1 Biomechanical analysis

#### 2.1.1 Specimens preparation

**FIGURE 1:**
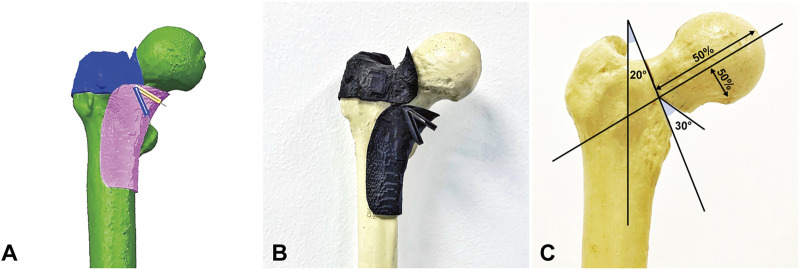
Creation of Pauwels Ⅲ femoral neck fracture. **(A)** Simulation of comminution 70° Pauwels Ⅲ femoral neck fractures with 30° distal wedge *via* Unigraphics NX 12.0 software. **(B)** Osteotomy jig made by 3D printing technology. **(C)** The osteotomy line passed through the midpoint of the femoral neck axis formed a 70° angle with the horizontal line and removed a 30° wedge fragment to eliminate medial support.

**FIGURE 2:**
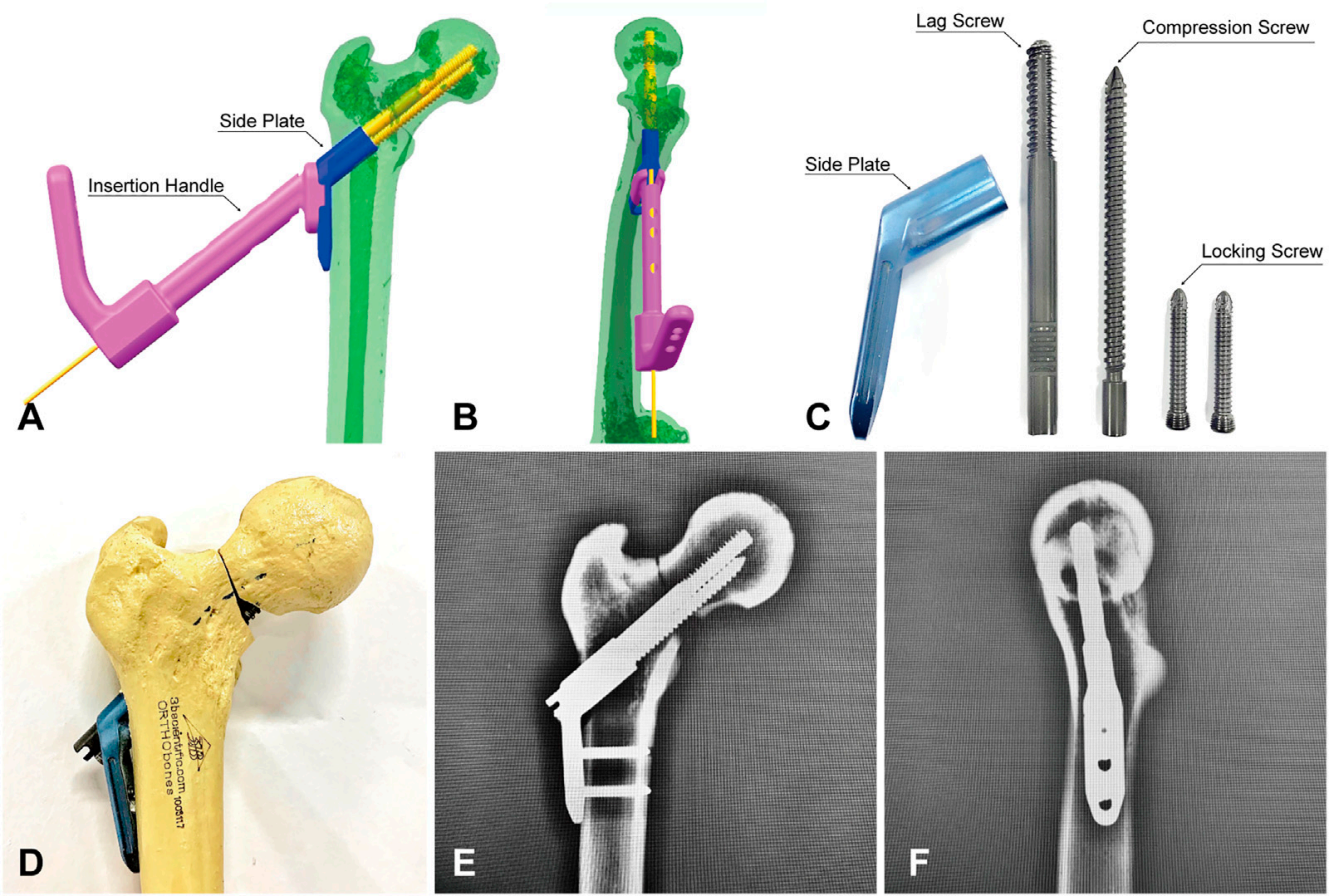
Fixation of Pauwels Ⅲ femoral neck fractures with IHS. **(A,B)** Simulation of IHS implantation in Unigraphics NX 12.0 software. **(C)** Components of IHS internal fixation. **(D)** Fixation with IHS. **(E,F)** anteroposterior and lateral fluoroscopic views after IHS fixation.

**FIGURE 3:**
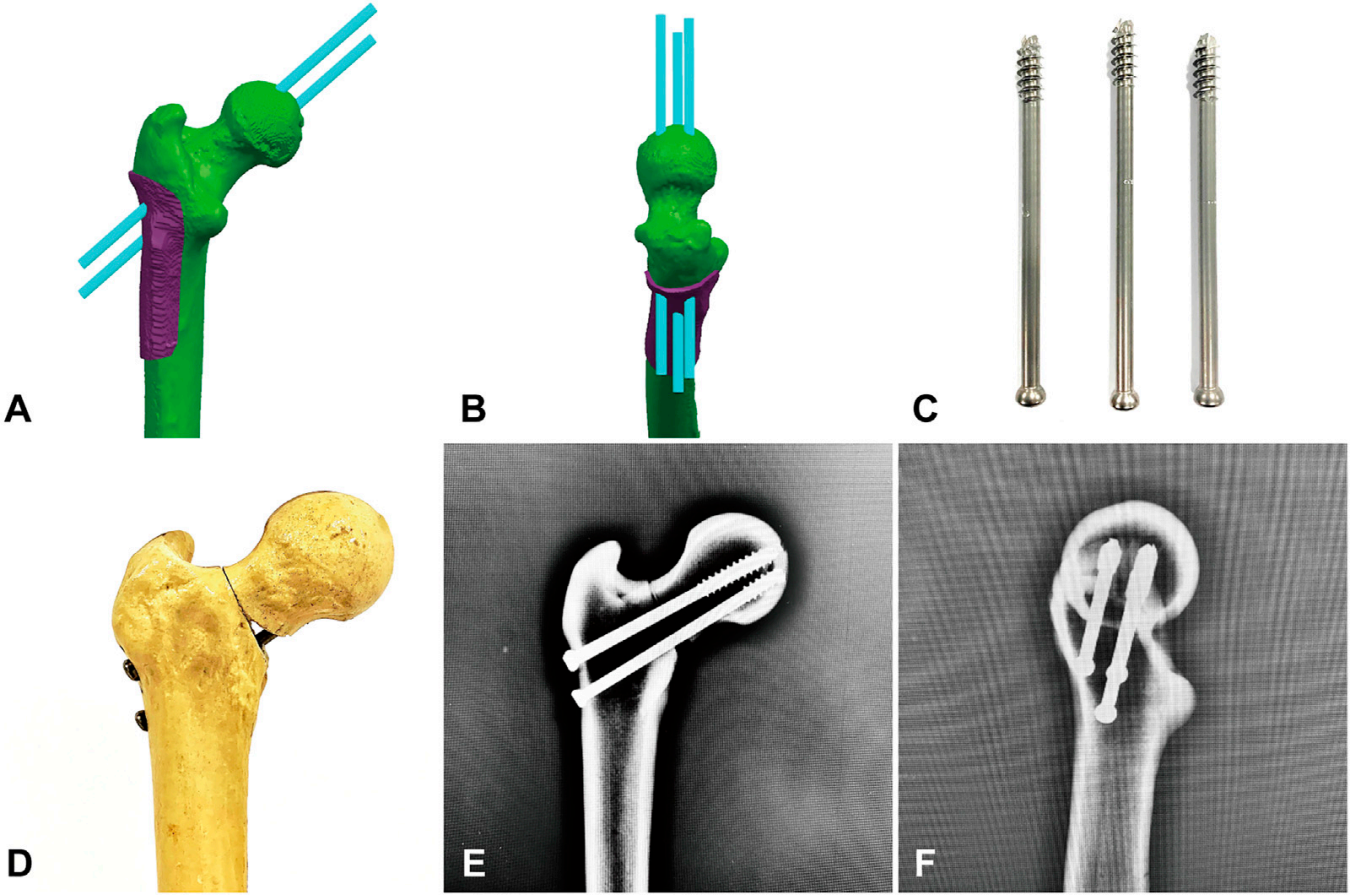
Fixation of Pauwels Ⅲ femoral neck fractures with ITCS. **(A,B)** Simulation of ITCS implantation in Unigraphics NX 12.0 software. **(C)** Components of ITCS internal fixation. **(D)** Fixation with ITCS. **(E,F)** anteroposterior and lateral fluoroscopic views after ITCS fixation.

Twenty composite femurs (Kim et al., 2022) (#1005117, ORTHObones, 3B Scientific, Hamburg, Germany) were randomly assigned to two groups: Group IHS (*n* = 10) fixed with IHS (Waston Medical, Changzhou, Jiangsu, China) and Group ITCS (*n* = 10) fixed with ITCS (Waston Medical, Changzhou, Jiangsu, China). Before osteotomy, all specimens were predrilled for subsequent anatomical reduction. The osteotomy line passed through the midpoint of the femoral neck axis formed a 70° angle with the horizontal line and removed a 30° distal wedge fragment to eliminate medial support (Stoffel et al., 2017; Knobe et al., 2018). We perform the osteotomy line in computer-aided design (CAD) Unigraphics NX 12.0 software beforehand and 3D printed custom cutting jig accordingly (Wang et al., 2022). Subsequently, a handsaw was used for osteotomy according to the custom-designed cutting jig (Figure 1). In Group IHS, the dynamic combined interlocking screws were inserted center–center in both anteroposterior and lateral views over one guidewire by using a set of dedicated instruments, and the two-hole 127° side plate was fixed to the femoral shaft by two 5.0 mm locking screws (Figure 2). In Group ITCS, three 7.3 mm partial thread cannulated screws were inserted into the femoral head in a standard inverted triangular configuration through cannulated screws guiding jig, resting the inferior screw on the calcar, and the anterosuperior and posterosuperior screws were close to the femoral neck cortex (Figure 3). Surgical simulations were carried out in Unigraphics NX 12.0 software to determine the appropriate length and position of the internal fixation, prior to fracture creation. All fixation procedures were performed by an experienced orthopedic trauma surgeon under the guidance of C-arm fluoroscopy according to the manufacturer’s guidelines. Each specimen was cut to a length of 25 cm, measured from the most superior point of the femur, and placed in a self-made metal cylinder tube potted with anchoring cement polymethylmethacrylate (PMMA) to satisfy the strength requirement of the biomechanical experiment (Bliven et al., 2020).

#### 2.1.2 Biomechanical protocol

**FIGURE 4:**
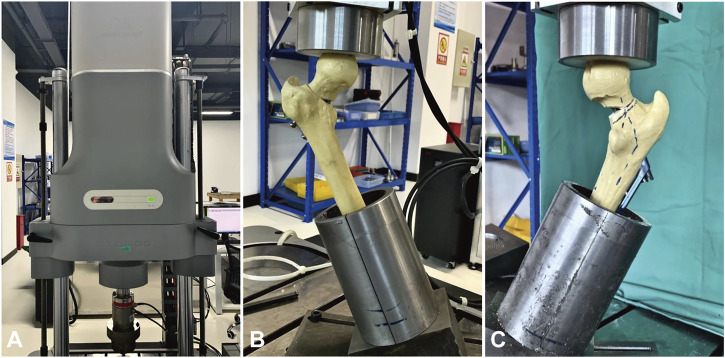
During the axial and cyclical compression tests, the specimen was installed in 25° adduction to simulate single leg stance. **(A)** Biomechanical testing was performed on a servo-hydraulic material testing system (Instron10000, Norwood, MA, United States). **(B)** Biomechanical test of Group IHS. **(C)** Biomechanical test of Group ITCS.

Biomechanical testing was performed on a servo-hydraulic material testing system (Instron10000, Norwood, MA, United States). All specimens were installed in 25° adduction of the femoral shaft (Figure 4) and loaded along the machine axis to simulate a single-leg stance (Schaefer et al., 2015; Kuan et al., 2016). A spherically shaped PMMA shell cup was attached to the machine actuator as a load mediator to transmit the pressure on the femoral head.

In the axial compression test, five synthetic femurs of Group IHS and five synthetic femurs of Group ITCS were tested for quasi-static ramped compression loading at the loading rate of 2 mm/min (Zhang et al., 2018). Machine data in terms of axial load and displacement were recorded *via* the servo-hydraulic material testing system. The failure of the specimen was defined as loosening, cutout, or breaking of internal fixation, fracture of synthetic femurs, the recorded displacement of 5 mm, and a sudden decrease in the recorded axial load (Zhang et al., 2018; Zhang et al., 2020).

In the cyclical compression test, the other five synthetic femurs of Group A and Group B were tested for double-peaked physiological compression cyclical loading (Bergmann et al., 2001)at 2 Hz for 10,000 cycles. The 10,000 loading cycles correspond to the number of steps walked at 4–6 weeks postoperatively, which is the expected period for preliminary fracture healing (Nuchtern et al., 2014; Steffensmeier et al., 2022). Keeping the valley load of each cycle at a constant level of 200 N and the peak load at 700 N to simulate partial weight-bearing after internal fixation of femoral neck fractures (Nowotarski et al., 2012). The ascending linear slope of the load-displacement curve in the axial compression test was defined as the axial stiffness of the bone–implant construct. The maximum failure load in the axial compression test and the max interfragmentary displacement during cyclic loading were recorded.

### 2.2 Clinical evaluation

#### 2.2.1 Inclusion and exclusion criteria

After obtaining the Institutional Review Board approval, we performed a retrospective study that enrolled 53 patients with Pauwels Ⅲ femoral neck fractures who underwent closed reduction and internal fixation with the IHS or ITCS in our trauma center from January 2020 to January 2021. Inclusion criteria were: the time interval from injury to operation < 3 weeks, Age > 18 years and <65 years, isolated Pauwels Ⅲ femoral neck fractures, and adequate radiographic and clinical follow-up. Exclusion criteria included: pathological femoral neck fractures, patients with previous hip fractures, and open fractures. There were 21 patients with Pauwels Ⅲ femoral neck fractures treated with IHS and made up Group IHS. Of 32 patients treated with ITCS, 21 patients were matched for age and sex with adequate radiographic and clinical follow-up, comprising Group ITCS.

#### 2.2.2 Surgical method

All patients with femoral neck fractures were evaluated preoperatively by an anesthesiologist. After medical contraindications were eliminated, the surgery was performed immediately by a senior trauma orthopedic surgeon. After general anesthesia, closed reduction was performed on the fracture traction table in the supine position. The C-arm is routinely placed between the legs to observe the quality of the fracture reduction and the position of the implant (Zhang and Tang, 2022). If anatomical position is difficult to obtain after three attempts, the Gotfried positive buttress reduction (Huang et al., 2020) would be adopted for some patients. In Group IHS, a straight lateral incision of approximately 4 cm was made in the proximal femur, directly across the fascia to the bone. In both AP and lateral views, a 3.0 mm guidewire was inserted along the center of the femoral neck to a depth of 5 mm beneath the surface of the femoral head cartilage. After reaming along the guidewire, a two-hole 127 degrees side plate and the dynamic combined interlocking screws were inserted into the femoral neck. The fracture gap was eliminated by the IHS unique combination compression mechanism that drove the semi-threaded lag screw when twisting the full-threaded compression screw in. Subsequently, the side plate was fixed to the femoral shaft by two 5.0 mm locking screws. In Group ITCS, three guide wires forming an inverted triangle configuration were inserted into the femoral head approximately 5 mm from the subchondral bone in both AP and lateral views (Tang et al., 2021). After ensuring the proper position, three 7.5 mm semi-threaded lag cannulated screws were inserted along the guide wires. Dissipation of the fracture gap during twisting of the cannulated screws.

#### 2.2.3 Perioperative management

Intravenous antibiotics were administered within 24 h after surgery to prevent infection. Oral rivaroxaban for 35 days postoperatively to prevent lower extremity deep vein thrombosis (Falck-Ytter et al., 2012). All patients were required to follow-up at 6 weeks, 3 months, and 6 months postoperative in the outpatient clinic for review, and every 6 months afterward. Patients in both groups were instructed to be non-weight bearing with crutches until 6 weeks postoperatively when partial weight bearing was started. Gradually increase the frequency and weight of partial weight-bearing exercises to full weight-bearing 3 months postoperatively (Dong et al., 2019).

#### 2.2.4 Data collection

Demographic, clinical, and radiographic data were obtained from the patient medical record. Demographic data included age, gender, and side of injury. Clinical information included mechanism of injury, time to surgery, implants used, operating duration, intra-operative blood loss, number of intra-operative fluoroscopies, length of hospital stay, fracture union time, Harris Hip Score (HHS), and the scores of Visual Analogue Scale (VAS) at 3-month and 6-month follow-up. Admission radiographs were analyzed for the Pauwels angle. Intra-operative and post-operative radiographs were analyzed to assess the quality of fracture reduction, implant placement, and complications such as nonunion, avascular necrosis, and femoral neck shortening at 12-month follow-up.

### 2.3 Statistical analysis

SPSS 25.0 (IBM, Armonk, NY, United States) was utilized for data analysis. Data were expressed as mean ± standard deviation or number (%). The count data were compared by a chi-squared test. Kolmogorov Smirnov test was used to analyze the normality of the distribution of continuous variables. Continuous variables of a normal distribution with homogeneous variance were compared between groups by *t*-test. Mann Whitney *U* test was used to compare continuous variables of a normal distribution with inhomogeneous variance and continuous variables not in line with normal distribution. All tests were two-tailed and assessed at the 5% significance level.

## 3 Results

### 3.1 Biomechanical analysis

**FIGURE 5:**
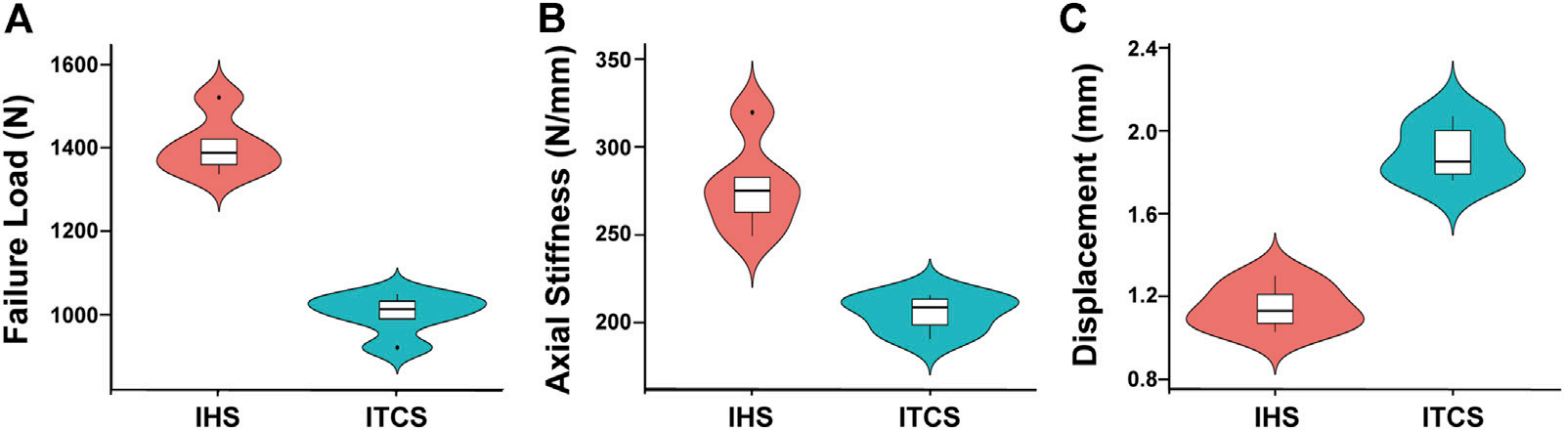
Biomechanical experimental data in Group IHS and Group ITCS. **(A)** Failure load during the quasi-static loading test of each group. **(B)** Axial stiffness during the quasi-static loading test of each group. **(C)** Interfragmentary displacement during the double-peaked physiological compression cyclic loading test of each group.

All specimens in the two groups survived in the axial compression and cyclical compression test without evidence of failure. The biomechanical data in terms of axial stiffness, failure load during the quasi-static loading test, and interfragmentary displacement during the double-peaked physiological compression cyclic loading test were shown in Figure 5. The mean axial stiffness was significantly higher for Group IHS (277.80 ± 26.58 N/mm) *versus* Group ITCS (205.33 ± 10.46 N/mm), *p* < 0.05. The mean maximum loading failure in Group IHS performed significantly better than that in Group ITCS (1,400.48 ± 71.60 N *versus* 996.76 ± 49.73 N, *p* < 0.05). The interfragmentary displacement of the cyclic loading test for Groups IHS and Group ITCS was 1.15 ± 0.11 mm and 1.89 ± 0.14 mm, respectively, *p* < 0.05.

### 3.2 Clinical analysis

**FIGURE 6:**
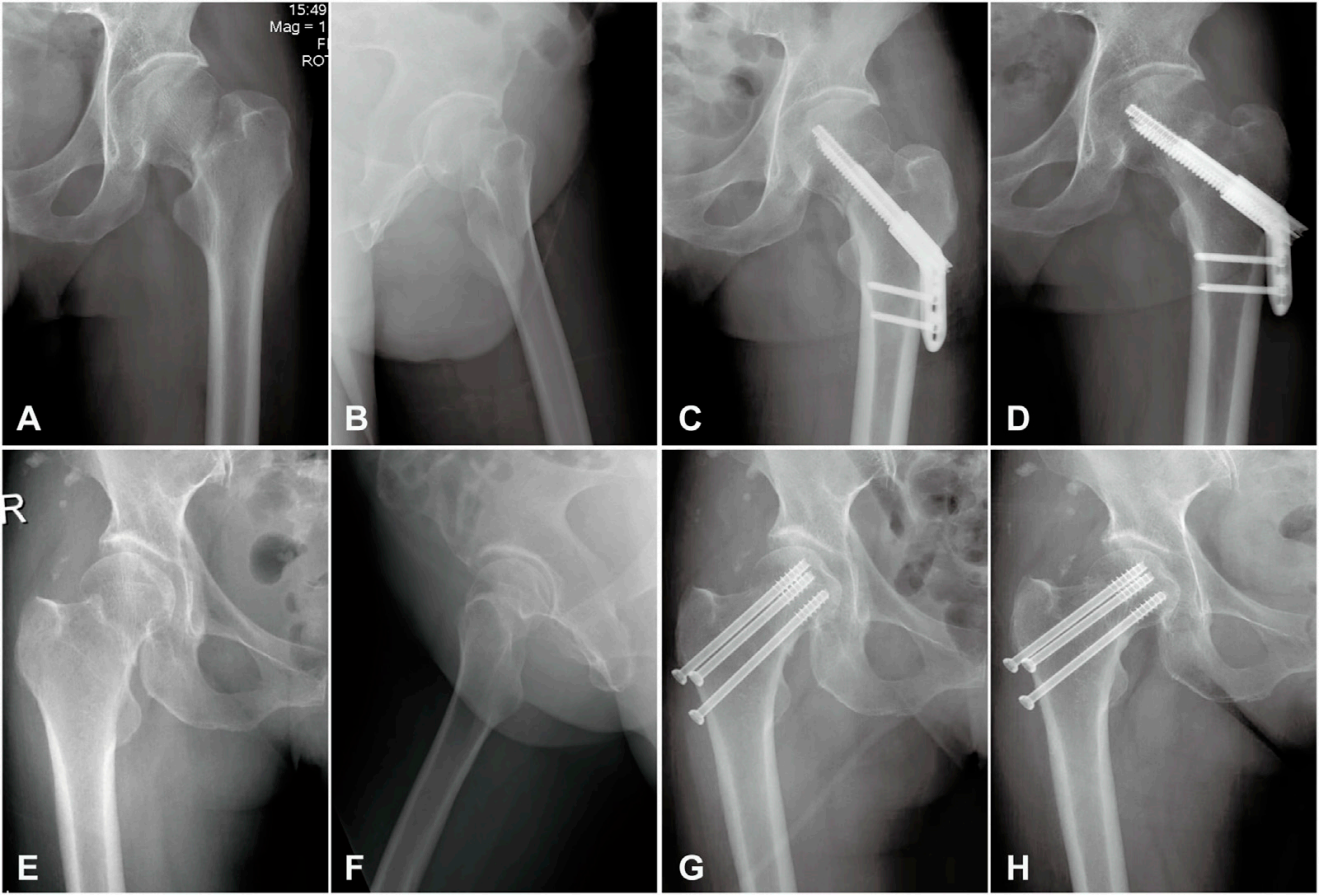
Radiographs of group IHS and group ITCS. **(A,B)**: Preoperative anteroposterior and lateral radiographs of a 44-year-old male with Pauwels Ⅲ femoral neck fracture. **(C)**: Anteroposterior radiographs of this patient fixed with the IHS technique at 1 day after operation. **(D)**: Anteroposterior radiographs of this patient fixed with the IHS technique at 3-month follow-up. **(E,F)**: Preoperative anteroposterior and lateral radiographs of a 56-year-old female with Pauwels Ⅲ femoral neck fracture. **(G)**: Anteroposterior radiographs of this patient fixed with the ITCS technique at 1 day after operation. **(H)**: Anteroposterior radiographs of this patient fixed with the ITCS technique at 3-month follow-up.

There were 21 matched patients (9 males and 12 females) in both Group IHS and Group ITCS. All patients in both groups received at least 1 year of clinical follow-up and typical caseswere shown in Figure 6. No significant difference was found between the two groups in terms of age, gender, side of injury, mechanism of injury, Pauwels angle, time to surgery, intraoperative blood loss, and length of hospital stay between the two groups, *p* > 0.05. Patients who underwent IHS treatment had shorter operating duration (51.43 ± 8.08 min *versus* 60.24 ± 10.43 min, *p* < 0.05) and fewer intraoperative fluoroscopic views (18.81 ± 3.82 times *versus* 24.67 ± 4.80 times, *p* < 0.05), as illustrated in Table 1.

**TABLE 1:** Comparison of baseline data and perioperative outcomes between the two groups.

Variables	IHS	ITCS	Statistical value	*p* value
Age (years)	52.86 ± 7.86	53.48 ± 7.12	t = -0.267	0.790
Gender (%)				
Male	42.86%	42.86%	χ2 = 0.000	1
Female	57.14%	57.14%		
Fracture side (%)				
Left	57.14%	52.38%	χ2 = 0.096	0.757
Right	42.86%	47.62%		
Pauwels angle (°)	64.39 ± 6.94	62.39 ± 7.56	t = 0.895	0.376
Injury mechanism (%)				
Low-energy trauma	47.62%	57.14%	χ2 = 0.382	0.537
High-energy trauma	52.38%	42.86%		
Time to surgery (days)	1.67 ± 0.58	1.71 ± 0.72	Z = -0.084	0.933
Operating duration (minutes)	51.43 ± 8.08	60.24 ± 10.43	t = -3.06	0.004
Intraoperative blood loss (ml)	56.19 ± 13.24	49.33 ± 8.54	t = 1.995	0.054
Fluoroscopy frequency (times)	18.81 ± 3.82	24.67 ± 4.80	t = -4.378	0.000
Length of hospital stay (days)	5.95 ± 1.50	6.33 ± 1.68	Z = -0.771	0.441

There were no significant differences between the two groups in terms of fracture union time and the scores of VAS at 3-month and 6-month follow-up. The HHS was 72.14 ± 5.76 and 86.62 ± 5.01 in Group HIS, and was 67.29 ± 5.27 and 81.76 ± 5.13 in Group ITCS at 3-month and 6-month follow-up, respectively, *p* < 0.05, indicating that IHS fixation facilitated the recovery of postoperative hip function. Concerning postoperative complications, there was no significant difference in the occurrence of nonunion and avascular necrosis between the two groups. Nevertheless, femoral neck shortening was observed in both groups. The magnitude of femoral neck shortening was significantly lower in Group IHS than in Group ITCS (4.80 ± 1.03 mm *versus* 5.56 ± 1.21 mm, *p* < 0.05), as illustrated in Table 2.

**TABLE 2:** Comparison of f function outcomes and postoperative complications between the two groups.

Variables	IHS	ITCS	Statistical value	*p* value
Fracture union time (month)	3.32 ± 0.40	3.45 ± 0.43	Z = -0.904	0.366
VAS				
3-month follow-up	2.67 ± 1.02	3.05 ± 1.53	Z = -0.644	0.519
6-month follow-up	1.62 ± 0.74	1.95 ± 1.50	Z = -0.499	0.618
HHS				
3-month follow-up	72.14 ± 5.76	67.29 ± 5.27	*t* = 2.581	0.007
6-month follow-up	86.62 ± 5.01	81.76 ± 5.13	*t* = 3.103	0.004
Complication				
Necrosis (%)	0%	4.80%	χ2 = 1.024	1
Nonunion (%)	0%	4.80%	χ2 = 1.024	1
Femoral neck shortening (mm)	4.80 ± 1.03	5.56 ± 1.21	*t* = -2.194	0.034

## 4 Discussion

With the increase of high-energy trauma such as traffic accidents and high-fall injuries, the incidence of femoral neck fractures in young adults has increased significantly, predominantly vertical Pauwels Ⅲ femoral neck fractures. The optimal selection of internal fixation for Pauwels Ⅲ femoral neck fractures remains controversial due to the high probability of femoral head necrosis, fracture nonunion, and femoral neck shortening. A previous expert opinion survey of the Orthopaedic Trauma Association’s (OTA) membership on the treatment of high-angle vertical femoral neck fractures in young adult patients showed that 46% of experts were unconvinced that their implant was supported by the literature (Luttrell et al., 2014). A 17-item survey completed by 540 surgeons from the Canadian Orthopaedic Association, the Orthopaedic Trauma Association, and attendees at an international fracture course revealed the lack of consensus on femoral neck fracture fixation in young people and there is an equal divide in preference between multiple cannulated screws and angular stable construct such as IHS(Slobogean et al., 2015).

To the best of our knowledge, the present study was the first study to evaluate the biomechanical and clinical evaluation of IHS and ITCS fixation on Pauwels Ⅲ femoral neck fractures. In our biomechanical experiment, we demonstrated that IHS had a significant benefit in terms of construct stiffness and protection from fracture displacement, compared with ITCS. Generally, the femoral neck fracture is fixed with ITCS according to the three-point principle (Bout et al., 1997). However, in the article by Collinge CA et al. (Collinge et al., 2014), major femoral neck comminution (>1.5 cm in any dimension) was identified in 96% of Pauwels Ⅲ femoral neck fractures cases, the same as in our chosen osteotomy model, resulting in insufficient stability of the second point in the three-point support for cannulated screws fixation.

Severe shortening of the femoral neck is associated with decreased gait velocity, impaired physical function, and the incidence of reoperation (Felton et al., 2019). IHS had better resistance to fracture micromotion and provided marked clinical benefit in terms of maintaining reduction, and femoral neck shortening in this small clinical series, which may be the reason for the significant difference in postoperative hip function between the two groups. As the successful initiation of fracture healing requires appropriate reduction and stabilization, the solid mechanical environment in the initial stages is one of the most important factors in achieving successful patient outcomes (Dong et al., 2019).

The combined interlocking screws of IHS are compressed immediately to reduce the fracture gap during operation and limited dynamic sliding compression after the operation following the mechanism of fracture healing (Einhorn and Gerstenfeld, 2015). The particular compression mechanism of the combined interlocking screws avoids the Z-effect phenomenon of two lag screws and the weak anti-rotation ability of a single screw (Strauss et al., 2007). The lateral locking compression plate of IHS fixed to the femoral shaft provided angular stability and resistance to shear force. The low radius of curvature of the lateral plate forms double line contact, reducing the contact area and decreasing compression of the periosteum as well as disrupting of the local blood supply. However, significant differences were unable to be demonstrated for nonunion and avascular necrosis of the femoral head, possibly due to the small sample size and inadequate follow-up duration.

In addition, our study found that the operative time was significantly shorter in the IHS group due to the relative manipulation simplicity. The IHS device is convenient to operate and inserted over one guide wire *via* a set of dedicated instruments. Operative time was generally considered as an influential factor associated with higher blood loss, longer anesthesia, higher infection rates, and overall higher postoperative complication rates (Cheng et al., 2018; Vazquez et al., 2021). The number of intraoperative fluoroscopies was fewer in patients who underwent IHS fixation. Mastrangelo et al. reported a significantly increased risk of cancer among orthopaedic surgeons exposed to long-term occupational radiation, with a cancer incidence rate of 29%, seven times higher than unexposed workers (Mastrangelo et al., 2005). Thus, it is essential to reduce the risk of radiation during surgical procedures (Yamashita et al., 2016; van Rappard et al., 2019).

However, there are several limitations in our biomechanical experiment and clinical research. To ensure the consistency of neck-shaft angle and bone density, we selected synthetic composite femurs as specimens rather than fresh-frozen human cadaveric femurs which are the gold standard for biomechanical experiment specimens (Windolf et al., 2009), and that may have a slight impact on the experimental results. We did not simulate the soft tissues surrounding the hip joint, such as muscles, joint capsules, and ligaments in the biomechanical experiment, which are important for stabilizing the structure and function of the hip joint (Fu et al., 2022). Our biomechanical experiments could not simulate the hip joint loading in all directions, such as A-P bending test which simulates the physiologic stress on the femoral head when rising from a seated position. Our clinical study was retrospective and observational design, and all data analyzed is subject to the quality of the data originally imported into the medical record, which might have resulted in methodological bias. Moreover, the relatively small patient size and short follow-up may result in the underestimation of the complication rate and prognosis.

## 5 Conclusion

In summary, our biomechanical experiment and the clinical study demonstrated IHS achieves better biomechanical support, shorter operative time, fewer intraoperative fluoroscopies, higher Harris hip score, and less femoral neck shortening due to its unique biological and biomechanical mechanisms, compared with inverted triangle cannulated screws. Thus, IHS is a feasible alternative to inverted triangle cannulated screws for the fixation of Pauwels Ⅲ femoral neck fractures. However, a long-term follow-up multicentre prospective controlled trial will be necessary to demonstrate the effectiveness of IHS in the fixation of Pauwels Ⅲ femoral neck fractures in the future.

## Data Availability

The original contributions presented in the study are included in the article/supplementary material, further inquiries can be directed to the corresponding authors.
